# EGPA: Eosinophilic granulomatosis with polyangiitis (Churg-Strauss syndrome) as a special presentation of chronic rhinosinusitis with nasal polyps (CRSwNP) 

**DOI:** 10.5414/ALX02475E

**Published:** 2024-03-21

**Authors:** Jan Hagemann, Martin Laudien, Sven Becker, Mandy Cuevas, Felix Klimek, Roya Kianfar, Ingrid Casper, Ludger Klimek

**Affiliations:** 1 Clinic for Ear, Nose and Throat Medicine, Mainz University Medical Center, Mainz,; 2 Clinic for Ear, Nose and Throat Medicine, University Medicine Kiel, Kiel,; 3 Clinic and Polyclinic for ENT Medicine, University Hospital Tübingen, Tübingen,; 4 Clinic and Polyclinic for ENT Medicine, Carl Gustav Carus University Hospital, TU Dresden, Dresden, and; 5 Center for Rhinology and Allergology, Wiesbaden, Germany

**Keywords:** eosinophils, EGPA, Churg-Strauss syndrome, eosinophilic granulomatosis with polyangiitis, vasculitis, ANCAs, CRSwNP

## Abstract

Introduction: Eosinophilic granulomatosis with polyangiitis (EGPA) was formerly known as Churg-Strauss syndrome. The condition is characterized by disseminated necrotizing vasculitis with extravascular granulomas associated with hypereosinophilia. The vasculitides affect small vessels and are associated with antineutrophil cytoplasmic antibodies (ANCAs) detectable in the blood. Distinguishing between type 2-mediated chronic airway inflammation such as chronic rhinosinusitis with nasal polyps (CRSwNP) without vasculitis can be clinically challenging and should be considered. Materials and methods: Immunological background, diagnosis, and therapy of EGPA were identified through literature searches in Medline, PubMed, as well as national and international studies (ClinicalTrials.gov) and the Cochrane Library. Human studies published up to and including 10/2023 on the topic were considered. Results: In cases of deteriorating general health with previously known eosinophilic inflammation of the upper and lower airways, EGPA and its interdisciplinary investigation should be considered. Various types of eosinophilic inflammation and syndromes must be considered differentially. Conclusion: Characterization of mucosal airway inflammation through biomarker determination is meaningful and occasionally makes the difference for targeted therapy.

## Eosinophilic airway inflammation in adults 

Chronic airway inflammation in adulthood can be divided into non-eosinophilic and eosinophilic. Some of these have their roots in childhood or adolescence and can be classified in the group of allergological diseases, which is often referred to as atopic march: atopic dermatitis is often followed by IgE-mediated allergic asthma and allergic rhinitis. Here too, the endotype is associated with eosinophil granulocytes. However, some others have their onset in adulthood and are characterized from the beginning by local or systemic hypereosinophilia (adult-onset). Prominent examples are (eosinophilic) chronic rhinosinusitis with nasal polyps ((e)CRSwNP), nonsteroidal anti-inflammatory drugs-exacerbated respiratory disease (N-ERD), allergic bronchopulmonary aspergillosis/mycosis (ABPA/ABPM), hypereosinophilic syndrome (HES), and eosinophilic granulomatosis with polyangiitis (EGPA) [1]. The joint occurrence of several of these diseases is common. 

The adult-onset eosinophilic phenotype of severe asthma was first described in a study from Great Britain [2]. Some groups report a gender imbalance in prevalence [3]. The typical minimum age is usually ~ 30 years on average (Table 1). This eosinophilic asthma probably affects between 20 and 40% of all severe asthma and is often associated with an increase in eosinophils in the blood. The supplementary measurement of expiratory nitric oxide (FeNO) is equally effective [4]. In contrast, the characterization of eosinophilia in CRSwNP is more inconsistent internationally, and recommendations are currently being developed by the European Association of Allergology and Clinical Immunology (EAACI). The detection of eosinophilic inflammation in paranasal sinus mucosa preparations is usually easy and is sufficient as a reliable indication of eCRSwNP according to the current recommendations of the European Society of Rhinology (ERS) [5]. 

Eosinophilic granulomatosis with polyangiitis (EGPA), formerly known as Churg-Strauss syndrome (CSS) [6] was first described by Churg and Strauss in 1951 as a rare disease characterized by disseminated necrotizing vasculitis with extravascular granulomas, occurring exclusively in patients with asthma and tissue eosinophilia [7, 8, 9]. The inflammatory vascular disease in EGPA is characterized by the fine tissue picture of penetration of the blood vessel walls with inflammatory cells. This leads to subsequent obstruction, formation of aneurysms, or even complete occlusion of the blood vessels. EGPA is therefore often classified as a primary systemic vasculitis [6]. The vascular inflammation disrupts the function of the affected organs. In 30 – 40% of patients, autoantibodies with antineutrophil cytoplasmic antibodies (ANCA) are detected in the blood, which is why EGPA is also classified as ANCA-associated vasculitis (AAV) [10]. However, the detection of ANCA alone is not decisive for the diagnosis. The ANCA-positive group among EGPA cases seems to be associated with a better prognosis, but also more involvement of the ENT area [11, 12]. 

In addition to inflammation of the vessel walls, granulomatous changes (granulomas) are also found near and far from the vessels, which particularly affect the lower airways, but can also occur in other organs such as the skin. The different and disease-defining processes of eosinophilic inflammation, granuloma formation, and vasculitis occur in parallel and independently of each other, which complicates diagnosis and characterization using biomarkers [1]. In addition to classic type 2 inflammatory profiles, IL-17, interferon-gamma and Th17 / Th1-mediated inflammatory pathways also exist [13, 14]. 

## Course of EGPA 

EGPA is traditionally divided into three phases [8, 15, 16]. The prodromal phase can begin months to years before the other phases and can last for a long time. EGPA is characterized by upper respiratory symptoms, asthma and general symptoms such as arthralgia, myalgia, malaise, fever, and weight loss [8]. The eosinophilic phase is characterized by peripheral eosinophilia and organ involvement, including pulmonary, cardiac, and gastrointestinal involvement. Migratory infiltrates in lung imaging are another hallmark of EGPA and one of the diagnostic criteria of the American College of Rheumatology. The vasculitic phase is manifested by constitutional symptoms such as fatigue, fever, and weight loss [8, 15]. 

The disease occurs preferably in adulthood. It is usually characterized by a history of bronchial asthma, often for many years, and chronic rhinosinusitis, usually with nasal polyps. Rhinitis and chronic sinusitis and/or otitis media with tympanic effusions are among the early manifestations of the disease. Olfactory disturbances are also observed, which are comparable to viral respiratory diseases [17], can lead to complete anosmia, and are usually persistent. 

Neurological involvement is observed in up to 60 – 70% of patients [18, 19]. Patients may present with multiplex mononeuritis or mixed sensory and motor peripheral neuropathy. The central nervous system is affected in 25% of cases with neurological involvement [8]. 

EGPA has some classic imaging findings. The most common radiologic manifestations in the lung are transient, bilateral, non-segmental areas of consolidation that occur in all lung segments [20, 21, 22]. Other abnormalities include small centrilobular nodules and, less commonly, larger nodules and interlobular septal thickening. Pleural effusion, a rare manifestation, has been described in a few patients [22]. 

The lung symptoms can be mild and yet be accompanied by very conspicuous lung shadows in the form of extensive infiltrates and, more rarely, round foci in the lungs on imaging. However, severe courses with shortness of breath and hemorrhage with bloody sputum are also possible, often combined with renal symptoms [18, 23, 24, 25] whereby other lung diseases should be considered in the differential diagnosis [26]. 

As the disease progresses, there is a massive increase in eosinophils in the blood with cell counts of > 1,000/μL in the blood and then vasculitis. Clinically, this usually manifests itself in severe general symptoms such as severe fatigue, fever, night sweats, unintentional weight loss and “rheumatic” complaints. These are often joint pains and aching muscles that change from day to day [18, 23, 24, 25]. Joint swelling is rare. Due to the preference for small vessels in vasculitis, signs of inflammation can occur in many organs, for example, in the lungs, heart, gastrointestinal tract, skin, kidneys, and nervous system. 

Pain in the kidney area due to swelling of the kidneys in the kidney capsule can occur. There are often indirect symptoms such as headaches with increased blood pressure, swelling of the eyelids and lower legs due to protein loss as a result of glomerulonephritis, as well as nausea, vomiting, and a severe feeling of illness when kidney failure occurs (uremia, edema). Dyspnea, arrhythmias, and chest pain may indicate cardiac involvement [7, 8, 9]. Abdominal pain, especially accentuated after eating, or persistent and sometimes bloody diarrhea may indicate involvement of the gastrointestinal tract in EGPA. Indications of involvement of the nervous system include numbness with discomfort in the tips of the fingers and feet (polyneuropathy), unsteady gait, and weakness due to concomitant myositis [7, 8, 9]. 

On the skin, punctiform hemorrhages (purpura) or extensive discoloration and, if medium-sized blood vessels are involved, ulceration with necrosis of large areas of skin and subcutaneous fatty tissue are found, for example on the fingers and toes (gangrene). Papular exanthema can also occur, and severe itching is sometimes reported [7, 8]. 

Cardiac involvement is the leading cause of death and poor prognosis in EGPA, occurring in 27 – 47% of EGPA cases [24]. Clinical manifestations include congestive heart failure, myocarditis, pericarditis, valvular abnormalities, myocardial ischemia, and arrhythmia [24, 27]. It occurs more frequently in ANCA-negative patients and correlates directly with eosinophilia in the blood [24]. 

## Diagnostics of EGPA 

The most reliable proof of EGPA is the removal of a tissue sample (biopsy) from an affected organ for histological examination with evidence of vasculitis and massive proliferation of eosinophils in the tissue. In the upper airway mucous membranes, however, it is usually only possible to detect eosinophilia, but not vasculitis, so that a biopsy from the nasal mucous membranes is not sufficient for the diagnosis of EGPA. When taking a sample from the nasal mucosa, the quality and localization of the sample must therefore be taken into account [10]. Eosinophilia can be found in nasal secretions and nasal cytology, peripheral sputum, bronchoalveolar lavage, and as tissue eosinophilia in histology [17, 19]. Eosinophilia in the peripheral blood of > 10% is one of the clinical criteria for the diagnosis of EGPA. p-ANCA/MPO-ANCA is detectable in the blood in ~ 30 – 40% of cases. 

Interdisciplinary collaboration between ENT specialists, ophthalmologists, rheumatologists, radiologists, and neurologists is recommended in order to accurately determine the activity and extent of the disease [28]. 

Imaging examinations such as chest X-ray or computer tomography of the lungs and magnetic resonance imaging (MRI) of the head including the paranasal sinuses are recommended. 

If cardiac involvement is suspected, echocardiography should be performed in addition to an ECG, and coronary angiography or an MRI of the heart should be performed if necessary. Pericardial effusion and myocarditis and/or cardiac arrhythmia may be present. 

Increased eosinophil cell counts or eosinophil markers can be detected in the bronchoalveolar lavage. Involvement of the gastrointestinal tract is detected by gastroscopy and colonoscopy with the removal of tissue samples. Laboratory tests often show an increase in erythrocyte sedimentation rate and CRP, anemia, leukocytosis, and thrombocytosis. High eosinophil counts of > 1,000/μL in the blood before therapy are often detected. Even if the disease relapses, the eosinophil count often rises again [28]. 

If the kidneys are involved, erythrocytes and protein are found in the urine. 

## EGPA therapy 

Severe courses of EGPA can initially be treated to induce remission with infusion therapy with cyclophosphamide in combination with cortisone. Once remission induction has been achieved, a remission-maintaining therapy phase with azathioprine or methotrexate can be maintained. Unfortunately, relapses are frequently and regularly observed. The main side effect of cyclophosphamide is an increased susceptibility to infections, which can occur with a reduced leukocyte count, which is why close blood count checks are recommended. 

During cyclophosphamide therapy, the amount drunk should be increased and, if necessary, a bladder protection preparation (Mesna, Uromitexan) should be taken, thus avoiding bloody cystitis and later bladder cancer. Azathioprine and methotrexate can cause blood count changes or an increase in liver function tests. At least half of patients with EGPA suffer a relapse during their lifetime. 

Alternatively, therapy with anti-interleukin-5 monoclonal antibodies (mepolizumab, benralizumab) [29, 30, 31] or rituximab (monoclonal antibodies against CD20-positive B lymphocytes), which should preferably be carried out by vasculitis centers experienced in the treatment of EGPA [28]. These monoclonal antibodies can significantly reduce eosinophil counts to the point of eosinopenia [32]. It should be noted here that the dose for mepolizumab in the EGPA indication is 300 mg Q4w, in contrast to the 3 times lower dose in the CRSwNP indication. 

Milder courses of the disease, especially without life-threatening organ manifestations, can be treated from the outset with azathioprine or methotrexate under careful monitoring. Co-treatment of patients with EGPA by a center experienced in diagnosis and treatment is recommended in German and international guidelines. 

## Discussion 

Eosinophilic airway inflammation often occurs in adulthood and can have various causes. The spread of biologics for the treatment of chronic inflammation and the determination of biomarkers associated with the therapy have increased awareness of eosinophilic diseases among healthcare professionals [5]. EGPA is a rare subgroup of eosinophilic airway inflammation, even within the severe asthmatic group. The diagnosis of EGPA should be considered in patients with asthma, chronic rhinosinusitis, and eosinophilia who experience clinical deterioration of the general condition and further complications of the nervous system, skin, lungs, or heart as well as kidneys [33, 34]. The vast majority (> 90%) of patients diagnosed with EGPA suffer from previously “long-standing” asthma, rarely showing seasonal exacerbations and worsening over time. There may also be a long-standing diagnosis of CRSwNP, and patients may even have undergone sinus surgery. In this respect, the presence of histopathological findings from the sinus surgery can also be deceptive, as this does not necessarily rule out the development of EGPA during the course. Other manifestations in the ENT specialty include otitis media. Eosinophilia (> 10% or > 1,500 cells per μL) is also observed in almost all patients with EGPA, although it can be masked by systemic glucocorticoids. 

An important differential diagnosis is HES, which has many features in common with EGPA, such as peripheral hyper-eosinophilia and tissue eosinophilia with organ dysfunction or damage [35]. The distinction between these two conditions has therapeutic and prognostic implications. For this reason, it has been suggested that patients who have asthma, eosinophils above 1,500/mm^3^, and systemic manifestations but no vasculitis in histologic specimens or ANCA be diagnosed as HES [35, 36, 37]. 

The diagnosis of EGPA should always be made on an interdisciplinary basis, which often proves difficult in practice due to time and resource constraints [16]. The diagnosis is not made on the basis of clear diagnostic criteria and scoring systems, but ideally by joint (interdisciplinary) detection of vasculitis, suggestive clinical signs, and ANCA detection in the blood. The currently valid criteria are only applicable in the case of histological evidence of small vessel vasculitis / vasculitis of medium-sized vessels (Table 2). New approaches for diagnostic criteria are currently still in the validation phase [38]. Despite the above-mentioned difficulties of biopsies from the upper airways, biopsies of new or abnormal findings are also advocated by recent guideline recommendations, as they can also rule out differential diagnoses and emphasize the activity of the inflammation over time. If vasculitis and p-ANCA are successfully detected in the blood, the group of possible diagnoses can be greatly restricted and focused on the group of vasculitides. The differentiation of EGPA from other small vessel vasculitides such as granulomatosis with polyangiitis (GPA) and microscopic polyangiitis is often uncomplicated due to differences in phenotypes and histology. However, granulomatosis with polyangiitis may occasionally present with peripheral or tissue eosinophilia, and a small proportion of EGPA patients may present with PR3-ANCA and a granulomatous and eosinophilic phenotype. 

## Conclusion 

Eosinophilic, chronic inflammation of the upper and lower airways often occur together in middle adulthood. The phenotypes can be very similar, which makes characterization according to pure biopsy or serology criteria difficult. Changes in the general clinical condition and disease activity of asthma or CRSwNP should invite more intensive, interdisciplinary diagnosis and clarification, as EGPA could be responsible. The detection of vasculitis from extra-respiratory organs and of p-ANCA in the blood are of great importance for the initiation of timely immunosuppressive therapy using cyclophosphamide, cortisone, or anti-IL-5(R) antibodies. 

CRSwNP patients with high blood eosinophilia in particular should not forego regular clinical checks. 

## Funding 

No funding was received for this work by any of the authors. 

## Conflict of interest 

J. Hagemann received grants from GSK, Sanofi, HAL Allergy, Novartis Pharma GmbH for lectures and consultations, outside the submitted work. 

M. Laudien received funds and/or fees from Olympus Deutschland GmbH, Olympus Europa SE & CO. KG, Novartis Pharma GmbH, Sanofi-Aventis Deutschland GmbH, Brainlab Sales GmbH, CSL Vifor, GlaxoSmithKline GmbH & Co. KG, Medtronic, Fiagon AG Medical Technologies, AEDA. He is board member of John Grube Foundation and chair of AG Rhinologie/Rhinochirurgie DGHNO. 

M. Cuevas has received honoraria from AstraZeneca, GSK, Sanofi, and Novartis, outside the submitted work. 

L. Klimek has received research grants and/or lecture fees from Allergy Therapeutics/ Bencard, UK/Germany; ALK-Abelló, Denmark; Allergopharma, Germany; ASIT Biotech, Belgium; AstraZeneca, Sweden, Bionorica, Germany; Biomay, Austria, Boehringer Ingelheim, Germany, Circassia, USA; Stallergene, France; Cytos, Switzerland; Curalogic, Denmark; HAL, Netherlands; Hartington, Spain; Lofarma, Italy; MEDA/Mylan, Sweden/USA; Novartis, Switzerland, Leti, Spain; ROXALL, Germany; GlaxoSmithKline (GSK), UK; Sanofi, France outside the submitted work and/or acted in an advisory capacity for the above-mentioned pharmaceutical companies. L. Klimek is editor of Allergo Journal and Allergo Journal International and he is the current President of AeDA (Ärzteverband Deutscher Allergologen) and Vice President of EAACI. 

R. Kianfar and F. Klimek report no conflicts of interest in connection with the present work. 

**Table 1.** Diseases involving the respiratory tract and hypereosinophilia [1].

	**Adult-onset eCRSwNP**	**EGPA**	**N-ERD**	**Adult-onset asthma**	**ABPA**
Prevalence (asthma/CRS patients)	3 – 10%	0.6%	5 – 10%	21 – 48%	2.5%
M : W	2 : 1	1 : 2	1 : 2	2 : 3	1 : 1
Appearance at the age of...	30 – 50	56	35 – 36	30 – 46	25 – 55
under 12	Rare	Rare	Rare	n.a.	Rare
over 60	Rare	Possible	Rare	Possible	Possible
Clinical peculiarity	Nasal polyps, loss of smell	Systemic vasculitis, granulomatosis	Salicylate intolerance	Rapid decrease in LuFu	Mucus plugs (bronchial tubes)
Biomarkers					
Blood-Eo	↑	↑↑↑	↑	↑↑	↑↑↑
Serum IgE (IU/mL)	Variable	100 – 300	Variable	Variable	1,000 – 10,000
FeNO	↑	↑	↑	↑	↑
Serum periostin	↑	↑→	↑	↑	↑↑
U LTE4	↑	↑↑	↑↑↑		
EETs/EETosis	++Lumen/tissue	Possible		++Lumen/tissue	++Lumen
Charcot-Leyden crystals	++Lumen/tissue	Possible		++Lumen/tissue	++Lumen
Other	CST-1, eotaxin-3, IgG4	ANCA, eotaxin-3, lösIL-2R	Mast cell/ tile marker	Reversibility in LuFu for diagnosis	
Response to biologics					
anti-IgE	+	+	+	+	+
anti-IL-5(R)	++	++	+	++	++
anti-IL-4R	++		++	++	
anti-TSLP	(+)			++	

eCRSwNP = eosinophilic chronic rhinosinusitis with nasal polyps; EGPA = eosinophilic polyangiitis with granulomatosis; N-ERD = non-steroidal-exacerbated respiratory disease; ABPA = allergic bronchopulmonary aspergillosis; LuFu = lung function test; FeNO = fractional expiratory NO; leukotriene E4 = LTE4; EETs/EETosis = extracellular eosinophil traps; ANCA = antineutrophil cytoplasmic antibody.

**Table 2.** The following criteria are only to be applied in the case of histological evidence of small vessel vasculitis.

**Clinical criteria**	Obstructive airway disease	+3
	Nasal polyps / CRSwNP	+3
	Mononeuritis multiplex	+1
**Laboratory and biopsy criteria**	Blood eosinophilia of >1,000 cells/µL or 1 × 10^9^/L (blood)	+5
	Extravascular, predominantly eosinophilic inflammation (biopsy)	+2
	cANCA + / anti-PR3 + (blood)	–3
	Hematuria	–1

A score of > 6 makes EGPA likely. CRSwNP = chronic rhinosinusitis with nasal polyps; cANCA = antineutrophil cytoplasmic antibody..

## References

[1] Asano K Ueki S Tamari M Imoto Y Fujieda S Taniguchi M Adult-onset eosinophilic airway diseases. Allergy. 2020; 75: 3087–3099. 33040364 10.1111/all.14620

[2] Haldar P Pavord ID Shaw DE Berry MA Thomas M Brightling CE Wardlaw AJ Green RH Cluster analysis and clinical asthma phenotypes. Am J Respir Crit Care Med. 2008; 178: 218–224. 18480428 10.1164/rccm.200711-1754OC PMC3992366

[3] Konno S Taniguchi N Makita H Nakamaru Y Shimizu K Shijubo N Fuke S Takeyabu K Oguri M Kimura H Maeda Y Suzuki M Nagai K Ito YM Wenzel SE Nishimura M Isada A Hattori T Shimizu K Yoshida T Distinct Phenotypes of Smokers with Fixed Airflow Limitation Identified by Cluster Analysis of Severe Asthma. Ann Am Thorac Soc. 2018; 15: 33–41. 28910142 10.1513/AnnalsATS.201701-065OC

[4] Lommatzsch M Criée CP de Jong CCM Gappa M Geßner C Gerstlauer M Hämäläinen N Haidl P Hamelmann E Horak F Idzko M Ignatov A Koczulla AR Korn S Köhler M Lex C Meister J Milger-Kneidinger K Nowak D Nothacker M [Diagnosis and treatment of asthma: a guideline for respiratory specialists 2023 – published by the German Respiratory Society (DGP) e. V.]. Pneumologie. 2023; 77: 461–543. 37406667 10.1055/a-2070-2135

[5] Fokkens WJ Viskens AS Backer V Conti D De Corso E Gevaert P Scadding GK Wagemann M Bernal-Sprekelsen M Chaker A Heffler E Han JK Van Staeyen E Hopkins C Mullol J Peters A Reitsma S Senior BA Hellings PW EPOS/EUFOREA update on indication and evaluation of Biologics in Chronic Rhinosinusitis with Nasal Polyps 2023. Rhinology. 2023; 61: 194–202. 36999780 10.4193/Rhin22.489

[6] Jennette JC Falk RJ Bacon PA Basu N Cid MC Ferrario F Flores-Suarez LF Gross WL Guillevin L Hagen EC Hoffman GS Jayne DR Kallenberg CG Lamprecht P Langford CA Luqmani RA Mahr AD Matteson EL Merkel PA Ozen S 2012 revised International Chapel Hill Consensus Conference Nomenclature of Vasculitides. Arthritis Rheum. 2013; 65: 1–11. 23045170 10.1002/art.37715

[7] Churg J Strauss L Allergic granulomatosis, allergic angiitis, and periarteritis nodosa. Am J Pathol. 1951; 27: 277–301. 14819261 PMC1937314

[8] Greco A Rizzo MI De Virgilio A Gallo A Fusconi M Ruoppolo G Altissimi G De Vincentiis M Churg-Strauss syndrome. Autoimmun Rev. 2015; 14: 341–348. 25500434 10.1016/j.autrev.2014.12.004

[9] Masi AT Hunder GG Lie JT Michel BA Bloch DA Arend WP Calabrese LH Edworthy SM Fauci AS Leavitt RY Lightfoot RW McShane DJ Mills JA Stevens MB Wallace SL Zvaifler NJ The American College of Rheumatology 1990 criteria for the classification of Churg-Strauss syndrome (allergic granulomatosis and angiitis). Arthritis Rheum. 1990; 33: 1094–1100. 2202307 10.1002/art.1780330806

[10] Laudien M Holl-Ulrich K Bioptische Diagnostik ANCAassoziierter Vaskulitiden im HNOTrakt. Z Rheumatol. 2023; 82: 53–55. 36542099 10.1007/s00393-022-01303-4 PMC9894962

[11] Comarmond C Pagnoux C Khellaf M Cordier JF Hamidou M Viallard JF Maurier F Jouneau S Bienvenu B Puéchal X Aumaître O Le Guenno G Le Quellec A Cevallos R Fain O Godeau B Seror R Dunogué B Mahr A Guilpain P Eosinophilic granulomatosis with polyangiitis (Churg-Strauss): clinical characteristics and long-term followup of the 383 patients enrolled in the French Vasculitis Study Group cohort. Arthritis Rheum. 2013; 65: 270–281. 23044708 10.1002/art.37721

[12] Cottin V Eosinophilic Lung Diseases. Clin Chest Med. 2016; 37: 535–556. 27514599 10.1016/j.ccm.2016.04.015

[13] Jakiela B Sanak M Szczeklik W Sokolowska B Plutecka H Mastalerz L Musial J Szczeklik A Both Th2 and Th17 responses are involved in the pathogenesis of Churg-Strauss syndrome. Clin Exp Rheumatol. 2011; 29: S23–S34. 21470488

[14] Tsurikisawa N Oshikata C Watanabe M Tsuburai T Kaneko T Saito H Innate immune response reflects disease activity in eosinophilic granulomatosis with polyangiitis. Clin Exp Allergy. 2018; 48: 1305–1316. 29908086 10.1111/cea.13209

[15] Mouthon L Dunogue B Guillevin L Diagnosis and classification of eosinophilic granulomatosis with polyangiitis (formerly named Churg-Strauss syndrome). J Autoimmun. 2014; 48-49: 99–103. 24530234 10.1016/j.jaut.2014.01.018

[16] Emmi G Bettiol A Gelain E Bajema IM Berti A Burns S Cid MC Cohen Tervaert JW Cottin V Durante E Holle JU Mahr AD Del Pero MM Marvisi C Mills J Moiseev S Moosig F Mukhtyar C Neumann T Olivotto I Evidence-Based Guideline for the diagnosis and management of eosinophilic granulomatosis with polyangiitis. Nat Rev Rheumatol. 2023; 19: 378–393. 37161084 10.1038/s41584-023-00958-w

[17] Klimek L Hagemann J Döge J Freudelsperger L Cuevas M Klimek F Hummel T Olfactory and gustatory disorders in COVID-19. Allergo J Int. 2022; 31: 243–250. 35755859 10.1007/s40629-022-00216-7 PMC9208356

[18] Moosig F L6. Eosinophilic granulomatosis with polyangiitis: future therapies. Presse Med. 2013; 42: 510–512. 23453508 10.1016/j.lpm.2013.01.006

[19] Woolnough K Wardlaw AJ Eosinophilia in Pulmonary Disorders. Immunol Allergy Clin North Am. 2015; 35: 477–492. 26209896 10.1016/j.iac.2015.05.002

[20] Lanham JG Elkon KB Pusey CD Hughes GR Systemic vasculitis with asthma and eosinophilia: a clinical approach to the Churg-Strauss syndrome. Medicine (Baltimore). 1984; 63: 65–81. 6366453 10.1097/00005792-198403000-00001

[21] Silva CI Müller NL Fujimoto K Johkoh T Ajzen SA Churg A Churg-Strauss syndrome: high resolution CT and pathologic findings. J Thorac Imaging. 2005; 20: 74–80. 15818205 10.1097/01.rti.0000155268.00125.de

[22] Worthy SA Müller NL Hansell DM Flower CD Churg-Strauss syndrome: the spectrum of pulmonary CT findings in 17 patients. AJR Am J Roentgenol. 1998; 170: 297–300. 9456932 10.2214/ajr.170.2.9456932

[23] Gibelin A Maldini C Mahr A Epidemiology and etiology of wegener granulomatosis, microscopic polyangiitis, churg-strauss syndrome and goodpasture syndrome: vasculitides with frequent lung involvement. Semin Respir Crit Care Med. 2011; 32: 264–273. 21674413 10.1055/s-0031-1279824

[24] Mahr A Moosig F Neumann T Szczeklik W Taillé C Vaglio A Zwerina J Eosinophilic granulomatosis with polyangiitis (Churg-Strauss): evolutions in classification, etiopathogenesis, assessment and management. Curr Opin Rheumatol. 2014; 26: 16–23. 24257370 10.1097/BOR.0000000000000015

[25] Sharma A Dogra S Sharma K Granulomatous Vasculitis. Dermatol Clin. 2015; 33: 475–487. 26143427 10.1016/j.det.2015.03.012

[26] Ciprandi G Schiavetti I Ricciardolo FLM Patients with asthma consulting an allergist differ from those consulting a pulmonologist. Allergo J Int. 2023; 32: 154–155.

[27] Sinico RA Bottero P Churg-Strauss angiitis. Best Pract Res Clin Rheumatol. 2009; 23: 355–366. 19508943 10.1016/j.berh.2009.02.004

[28] Caminati M Eosinophilic granulomatosis with polyangiitis onset in severe asthma patients on monoclonal antibodies targeting type 2 inflammation: Report from the European EGPA study group. Allergy. 2024 .; 79: 516–519. 37904674 10.1111/all.15934

[29] Cottu A Groh M Desaintjean C Marchand-Adam S Guillevin L Puechal X Beaumesnil S Lazaro E Samson M Taille C Durel CA Diot E Nicolas S Guilleminault L Ebbo M Cathebras P Dupin C Yildiz H Belfeki N Pugnet G Benralizumab for eosinophilic granulomatosis with polyangiitis. Ann Rheum Dis. 2023; 82: 1580–1586. 37550002 10.1136/ard-2023-224624

[30] Portacci A Campisi R Buonamico E Nolasco S Pelaia C Crimi N Benfante A Triggiani M Spadaro G Caiaffa MF Scioscia G Detoraki A Valenti G Papia F Tomasello A Scichilone N Pelaia G Crimi C Carpagnano GE Real-world characteristics of “super-responders” to mepolizumab and benralizumab in severe eosinophilic asthma and eosinophilic granulomatosis with polyangiitis. ERJ Open Res. 2023; 9: 00419-2023. 37908397 10.1183/23120541.00419-2023 PMC10613971

[31] Terrier B Jayne DRW Hellmich B Bentley JH Steinfeld J Yancey SW Kwon N Akuthota P Khoury P Baylis L Wechsler ME Clinical Benefit of Mepolizumab in Eosinophilic Granulomatosis With Polyangiitis for Patients With and Without a Vasculitic Phenotype. ACR Open Rheumatol. 2023; 5: 354–363. 37312233 10.1002/acr2.11571 PMC10349249

[32] Miranda J Plácido JL Amaral L A case of protracted eosinopenia after a single subcutaneous dose of benralizumab. Allergo J Int. 2022; 32: 1–2. 10.1007/s40629-022-00227-4 PMC9513006 36185337

[33] Andersen H Götz P Bremer JP Laudien M [Manifestation of eosinophilic granulomatosis with polyangiitis in the head and neck area over time taking systemic disease activity into consideration]. Z Rheumatol. 2018; 77: 928–937. 29569004 10.1007/s00393-018-0439-0

[34] Petersen H Götz P Both M Hey M Ambrosch P Bremer JP Holle J Moosig F Laudien M Manifestation of eosinophilic granulomatosis with polyangiitis in head and neck. Rhinology. 2015; 53: 277–285. 26363169 10.4193/Rhino14.074

[35] Schwaab J Lübke J Reiter A Metzgeroth G Idiopathic hypereosinophilic syndrome – diagnosis and treatment. Allergo J Int. 2022; 31: 251–256.

[36] Gotlib J World Health Organization-defined eosinophilic disorders: 2014 update on diagnosis, risk stratification, and management. Am J Hematol. 2014; 89: 325–337. 24577808 10.1002/ajh.23664

[37] Valent P Klion AD Horny HP Roufosse F Gotlib J Weller PF Hellmann A Metzgeroth G Leiferman KM Arock M Butterfield JH Sperr WR Sotlar K Vandenberghe P Haferlach T Simon HU Reiter A Gleich GJ Contemporary consensus proposal on criteria and classification of eosinophilic disorders and related syndromes. J Allergy Clin Immunol. 2012; 130: 607–612.e9. 22460074 10.1016/j.jaci.2012.02.019 PMC4091810

[38] Wechsler ME Akuthota P Jayne D Khoury P Klion A Langford CA Merkel PA Moosig F Specks U Cid MC Luqmani R Brown J Mallett S Philipson R Yancey SW Steinfeld J Weller PF Gleich GJ Mepolizumab or Placebo for Eosinophilic Granulomatosis with Polyangiitis. N Engl J Med. 2017; 376: 1921–1932. 28514601 10.1056/NEJMoa1702079 PMC5548295

[39] Grayson PC Ponte C Suppiah R Robson JC Craven A Judge A Khalid S Hutchings A Luqmani RA Watts RA Merkel PA 2022 American College of Rheumatology/European Alliance of Associations for Rheumatology Classification Criteria for Eosinophilic Granulomatosis with Polyangiitis. Ann Rheum Dis. 2022; 81: 309–314. 35110334 10.1136/annrheumdis-2021-221794

